# Neighborhood Deprivation and Suicide Among Adolescent and Young Adult Cancer Patients

**DOI:** 10.1002/cam4.71247

**Published:** 2025-09-26

**Authors:** Abhery Das, Cecily A. Byrne, Vanessa M. Oddo, Sidra Goldman‐Mellor, Sage J. Kim

**Affiliations:** ^1^ Health Policy & Administration, School of Public Health University of Illinois Chicago Chicago Illinois USA; ^2^ Cancer Health Equity and Career Development Program University of Illinois Chicago Chicago Illinois USA; ^3^ Department of Kinesiology and Nutrition University of Illinois Chicago Chicago Illinois USA; ^4^ Department of Public Health University of California Merced California USA

**Keywords:** adolescent and young adult, cancer, mental health, suicide, youth

## Abstract

**Background:**

In 2024, approximately 84,100 adolescents and young adults (AYAs) between 15 and 39 years old received a cancer diagnosis.

**Aims:**

Given their unique psychosocial, economic, and clinical stressors, we examined whether AYA cancer patients living in deprived neighborhoods have a higher risk of suicide when compared to those in the least deprived neighborhoods.

**Materials & Methods:**

Our sample comprised 486,374 AYA cancer patients from the Surveillance, Epidemiology, and End Results (SEER) dataset between 2006 and 2020. We use Cox proportional hazard models to test the relation between quintiles of neighborhood deprivation, from Q1 (most deprived) to Q5 (least deprived), and survival months until suicide mortality from the time of cancer diagnosis.

**Results:**

We find that AYA cancer patients living in more deprived neighborhoods (Q1, Q2, Q4) have a higher risk of suicide when compared to those in the least deprived neighborhoods (Q5) (Q1: HR—1.82 [1.14–2.90]; Q2: HR—1.95 [1.35–2.81]; Q4: HR—1.48 [1.05–2.07]).

**Discussion:**

Mental health services and monitoring from treatment through survivorship may support suicide prevention efforts for young cancer patients.

**Conclusion:**

Early identification of high‐risk AYA cancer patients living in deprived areas may help target suicide prevention interventions.

## 1 Introduction

In 2024, approximately 84,100 adolescent and young adults (AYAs) ages 15 through 39 received a cancer diagnosis [1]. AYAs have had the greatest increase in cancer incidence when compared to other age groups [2]. Although underexamined, early findings suggest that this may result from a lack of exercise, sleep, dietary quality, as well as cancer screening in young adults [3]. The most common cancers among AYAs include breast, thyroid, and testicular cancers, and melanomas [1]. AYAs with cancer have a higher prevalence of suicide at 17.68 per 100,000 population when compared to the AYA general population at 14.33 per 100,000 population [4].

Sociological theories on suicide, dating back to Durkheim, posit that social integration and networks within communities can protect against suicidality [5]. The Status Integration Theory suggests that suicide risk increases as the dissonance between an individual and their community grows [6]. Further, empirical work finds that neighborhood‐ and county‐level socioeconomic deprivation and social fragmentation contribute to suicide mortality [7, 8, 9]. One study suggests that perceptions of neighborhood deprivation, rather than objective measures of neighborhood quality, coincide with youth suicidality [10].

Among cancer patients, area‐level studies also find that those from socioeconomically deprived counties have a higher risk of suicide [11, 12, 13]. One study reports that cancer patients have a greater likelihood of suicide if they live in lower‐income or rural counties [12]. Other scholars document that, among prostate cancer patients, those living in counties with lower median household incomes have a higher risk of suicide [11]. Additionally, suicide rates are elevated among cancer patients living in counties with lower educational attainment and greater poverty or unemployment [13]. Given their unique clinical, psychosocial, and economic stressors [14], AYA cancer patients in deprived neighborhoods may also have a higher likelihood of suicide mortality.

Although important, previous work remains limited in several ways. First, studies do not focus on the extent to which neighborhood deprivation may affect suicide mortality among AYAs. AYAs may be in school, beginning careers, or starting families when receiving their cancer diagnoses [1, 4]. Maintaining such responsibilities with a cancer diagnosis may present particular challenges for AYAs, as they show a greater risk of unemployment, lower incomes, and the need for governmental income supplementation when compared to their cancer‐free peers [15]. Similarly, AYA cancer patients also have greater long‐term adverse health outcomes following treatment, such as cardiovascular disease and depression [16]. Living in neighborhoods with limited resources may not only exacerbate barriers to employment but also limit access to other social services, such as health care or housing, that would make integration during and after treatment easier for AYAs.

Second, previous work has primarily evaluated whether county‐level, as opposed to neighborhood‐level, deprivation corresponds with an increased risk of suicide among cancer patients [11, 12, 13]. County‐level differences in health outcomes may arise due to policies or economic structures. In contrast, neighborhood‐level factors may reflect how physical and social environments affect mental health among cancer patients [17]. Inequities in resource distribution can influence access to social services and healthcare resources [17, 18]. Physical disorder, safety, social cohesion, and institutional support may also vary by neighborhood [17, 18]. Combined, physical and social environments contribute to health outcomes within neighborhoods [17, 18]. Neighborhood characteristics have shown substantial impacts on physical and mental health outcomes [17, 18]. Importantly, neighborhood quality [19, 20], perceptions of disorder [21, 22, 23], built environment [24, 25], and accessibility coincide with depressive symptoms [26, 27], a significant risk factor for suicide [28].

We extend previous work by examining whether neighborhood deprivation corresponds with suicide among AYA cancer patients. We use the Surveillance, Epidemiology, and End Results (SEER) database with records for 486,374 AYA cancer patients from 22 registries between 2006 and 2020. We hypothesize that AYA cancer patients living in neighborhoods with greater deprivation will have a higher risk of suicide mortality. Our study provides insight into the upstream drivers of suicide mortality in a high‐risk population, as well as potential areas for community‐level interventions.

## 2 Methods

### 2.1 Study Population

The National Cancer Institute designates AYA cancer patients as those aged 15–39 years old [1]. The study population comprised 486,374 AYA cancer patients who were diagnosed with a first primary malignant tumor between January 1, 2006 and December 31, 2020. We extracted data from the Incidence—SEER Research Plus Limited‐Field Specialized Data (with Census Tract Attributes), 22 Registries (excluding Alaska, Illinois, and Massachusetts) (SEER 22) [29]. SEER 22 follows cancer patients from diagnosis until whichever comes first: death, last known date alive, or end of follow‐up by December 31, 2020 [30]. We excluded the following patients: (a) with unknown age, given our restriction to AYA cancer patients; and (b) those who did not have information on survival months. Of the 486,374 AYA cancer patients, 456 died by suicide or self‐inflicted injury during our study period. The 456 AYA cancer patients who died by suicide were then compared to the 485,918 who did not die by suicide and are still alive or have died from other causes.

### 2.2 Study Measures

As our outcome, we used survival months until mortality from suicide or self‐inflicted injury from the time of cancer diagnosis, among AYA cancer patients. SEER utilizes ICD‐10 codes for suicide or self‐inflicted injury cause of death, which comprise U03, X60‐X84, Y87.0.

As our exposure, we used quintiles of neighborhood deprivation for census tracts in which AYAs resided at the time of diagnosis, obtained from the SEER Specialized Databases [31]. SEER estimated neighborhood deprivation scores using factor analyses from census tract level variables [32], including: median household income, median house value, median rent, percent of individuals living below 150% of the federal poverty line, education index [33], percent working class, and percent unemployed [34, 35]. SEER then categorized scores into quintiles with equal populations in each quintile across the US. Quintile 1 (Q1) comprised census tracts with the greatest neighborhood deprivation (20th percentile or lower socioeconomic characteristics), and Quintile 5 comprised census tracts with the least neighborhood deprivation (80th percentile or higher socioeconomic characteristics). Quintiles for each census tract (from corresponding American Community Survey 5‐Year estimates) were then linked to patients' cancer diagnoses by year. All quintiles used the Decennial 2010 census tract boundaries [36].

Based on prior literature, covariates included individual‐level factors, including age, race/ethnicity, sex, cancer stage, primary AYA site [37, 38], and relationship status. Neighborhood‐level attributes included indicators for persistent poverty (20% or more of the population is below the federal poverty line in previous census measures) and urbanicity at the census tract level.

SEER provides de‐identified and publicly available data under a data use agreement with the National Institutes of Health. Researchers must request access to the SEER Specialized Database. The institution deemed this study exempt from review due to publicly available and de‐identified data [39].

### 2.3 Statistical Analysis

We estimated Hazard Ratios (HRs) using Cox proportional hazards regression to identify the risk of suicide death among AYAs with cancer while accounting for other causes of death or survival as competing risks. We first tested the proportional hazards assumption for each covariate using visual examinations of survival probability (Kaplan–Meier curves) and log‐rank tests to examine equality across strata of categorical variables. We verified that the overall Cox model satisfied the assumption of proportionality using the Schoenfeld method [40].

We then examined whether the risk of suicide death differed by quintile of neighborhood deprivation score using Cox proportional hazards regression while adjusting for individual‐ and neighborhood‐level covariates. We utilized the least deprived neighborhoods (Q5) as the reference group for the exposure of interest. We conducted a sensitivity analysis in which we removed all individuals who died from cancer and other health conditions from our sample. This test compared those who died by suicide to only those who are still alive. We aimed to isolate the impact of neighborhood deprivation and on suicide, as opposed to cancer and other health conditions that may also worsen due to a lack of resources and care from area‐level deprivation.

We used Stata 16 (StataCorp LLC, College Station, TX, USA) for all statistical analyses.

## 3 Results

Table 1 details the demographic and neighborhood characteristics of AYA cancer patients. Most AYA cancer patients in our sample identify as non‐Hispanic (NH) White (54.7%), male (61.1%), and in the 35–39 age group (38.7%). Most AYA cancer patients have carcinomas (52.7%) and cancers in the localized region (52.7%). Survival months average 70.5 months for AYA cancer patients. The majority of AYA cancer patients live in the least deprived neighborhoods (Q5: 25.7%), in urban areas (90.5%), and in neighborhoods that are not persistently impoverished (89.1%).

**TABLE 1:** Individual and neighborhood characteristics of adolescent and young adult (AYA) cancer patients overall and those who died by suicide or self‐inflicted injury from 22 registries in the United States, 2006–2020.

Characteristic	AYAs with cancer who died by suicide *N* = 456	AYAs with cancer *N* = 486,374
	*N* (%)	*N* (%)
Individual characteristics		
Race/ethnicity (%)		
NH White	335 (73.5)	265,888 (54.7)
NH Black	26 (5.7)	50,606 (10.4)
Hispanic	61 (13.4)	120,779 (24.8)
Other	34 (7.50)	49,101 (10.1)
Sex (%)		
Female	181 (39.7)	296,965 (61.1)
Male	275 (60.3)	189,409 (38.9)
Age group (%)		
15–19	26 (5.7)	32,396 (6.7)
20–24	55 (12.1)	52,338 (10.8)
25–29	69 (15.1)	84,883 (17.5)
30–34	91 (20.0)	128,691 (26.5)
35–39	215 (47.2)	188,066 (38.7)
Relationship status (%)		
Single (never married)	177 (53.47)	181,117 (50.1)
Married (including common law)	122 (36.9)	160,446 (44.4)
Other	32 (9.7)	19,980 (5.5)
Stage (%)		
In situ/localized	215 (54.2)	189,784 (52.7)
Regional	102 (25.7)	95,788 (26.6)
Distant	80 (20.2)	74,803 (20.8)
Tumor site (%)		
Leukemias and related disorders	31 (6.8)	31,654 (6.5)
Lymphomas	66 (14.5)	54, 660 (11.2)
CNS, intracranial, intraspinal	19 (4.2)	19,522 (4.0)
Sarcomas	19 (4.2)	22,043 (4.5)
Blood and lymphatic vessel	3 (0.7)	4173 (0.9)
Nerve sheath	2 (0.4)	1024 (0.2)
Gonadal and related tumors	73 (16.0)	50,382 (10.4)
Melanomas	39 (8.6)	40, 671 (8.4)
Carcinomas	196 (43.0)	256,307 (52.7)
Miscellaneous neoplasms	1 (0.2)	1740 (0.4)
Unspecified malignant neoplasms	7 (1.5)	4168 (0.9)
Survival months (mean)	54.7	70.5
Neighborhood characteristics		
Neighborhood deprivation		
Quintile 1 (most deprived)	74 (16.4)	81,473 (17.2)
Quintile 2	92 (20.4)	81, 517 (17.2)
Quintile 3	86 (19.0)	87,504 (18.5)
Quintile 4	108 (23.9)	101,826 (21.5)
Quintile 5 (least deprived)	92 (20.4)	121,889 (25.7)
Persistent poverty		
No	416 (91.2)	432,771 (89.1)
Yes	40 (8.8)	53,185 (10.9)
Urbanicity		
Urban	401 (87.9)	439,826 (90.5)
Rural	55 (12.1)	46,110 (9.5)

Abbreviation: CNS, central nervous system.

The majority of AYA cancer patients who died by suicide show similar demographic characteristics as the broader AYA group (Table 1). Most AYA cancer patients who died by suicide are NH white (73.5%), male (60.3%), in the 35–39 age group (47.2%), have carcinomas (43%), and cancers in a localized region (54.2%). However, this subgroup does not live as long, averaging 54.7 survival months. The largest proportion of AYA cancer patients who died by suicide live in the fourth quintile of neighborhood deprivation (23.9%), in urban areas (87.9%), and in neighborhoods that are not classified as persistently impoverished (91.2%).

**FIGURE 1:**
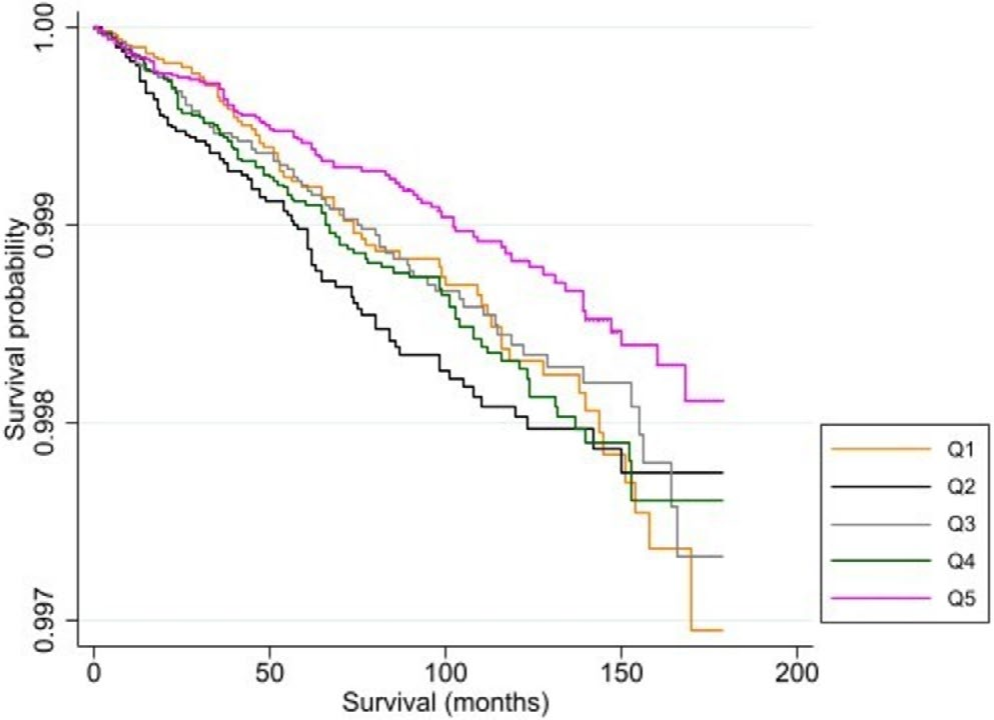
Survival probability of death by suicide or self‐inflicted injury among adolescent and young adult (AYA) cancer patients by quintile^1^ of neighborhood deprivation from 22 cancer registries in the United States, 2006–2020. *Log‐rank test: *p* < 0.05. ^1^Q1 (most deprived) − Q5 (least deprived).

Figure 1 shows the Kaplan–Meier curve with survival probability of death by suicide among AYA cancer patients by quintile of neighborhood deprivation. The quintiles do not appear entirely parallel; however, log‐rank tests verify differences in survival (*p* < 0.05) and the Schoenfeld method verifies the assumption of proportionality (Table S2 and Figure S1). In Figure 1, Q2 neighborhoods show the lowest probability of survival for most survival months; however, Q1 neighborhoods show the lowest probability of survival after approximately 150 months of survival. Q5 neighborhoods consistently show a higher probability of survival throughout the majority of survival months. Figures S2–S8 present survival probabilities for each of the covariates used in the model and results from their log‐rank tests. We utilized a threshold of *p* < 0.25 to initially screen variables using log‐rank tests to avoid excluding potentially important variables based on prior literature [41].

Table 2 shows the Cox proportional hazards results. We find that AYA cancer patients living in the most deprived neighborhoods (Q1) have a higher risk of dying by suicide when compared to the least deprived neighborhoods (Hazard Ratio (HR): 1.82, 95% Confidence Interval (CI): 1.14–2.90). We also see a greater risk of suicide among AYA cancer patients living in Q2 and Q4 of neighborhood deprivation, when compared to the least deprived neighborhoods: For Q2, the HR was 1.95 (95% CI: 1.35–2.82) and for Q4, HR was 1.48 (95% CI: 1.05–2.07). AYA cancer patients living in Q2 neighborhoods show the greatest risk of suicide, followed by those living in Q1 and then those living in Q4. AYA cancer patients living in Q3 of neighborhood deprivation also see a greater risk of suicide; however, our results do not reach conventional levels of statistical detection (HR: 1.37, 95% CI: 0.94–1.98).

**TABLE 2:** Cox proportional hazards model predicting risk of death by suicide or self‐inflicted injury as a function of quintiles of neighborhood deprivation among 265,767^a^ adolescent and young adult (AYA) cancer patients from 22 registries in the United States, 2006–2020.

Characteristic	Hazard ratio (95% CI)
Neighborhood deprivation	
Quintile 1 (most deprived)	1.82 (1.14–2.90)**
Quintile 2	1.95 (1.35–2.82)***
Quintile 3	1.37 (0.94–1.98)
Quintile 4	1.48 (1.05–2.07)**
Ref: Quintile 5 (least deprived)	—

^a^ Adjusted for individual and neighborhood‐level covariates.

* *p* < 0.1.

** *p* < 0.05.

*** *p* < 0.001.

Results for our Cox proportional hazards model (including covariates) are presented in Table S1. We report that NH Black, Hispanic, and Other race/ethnicities show a lower risk of dying by suicide when compared to non‐Hispanic Whites. Males, patients in the 35–39 age group, AYAs with cancers at a later stage, and those who live in rural neighborhoods have a higher risk of dying by suicide.

Our sensitivity test that removed all AYA cancer patients who died from cancer or other health conditions from our sample showed essentially the same results as our main analysis (Table S3).

## 4 Discussion

AYA cancer patients have a higher risk of suicide than their counterparts in the general population [4]. AYAs present a challenge due to their age, in which cancer diagnoses may interrupt major life experiences such as schooling, professional careers, or family formation [1, 4]. Neighborhood deprivation may contribute to the mental health consequences of such cancer diagnoses and treatment [17, 18]. We find that AYA cancer patients living in socioeconomically deprived neighborhoods show a greater risk of suicide when compared to those living in the least deprived neighborhoods.

Our findings cohere with previous theoretical and empirical work showing that area‐level socioeconomic deprivation and subsequent social fragmentation coincide with greater suicide mortality [5, 8, 9, 42, 43, 44]. Studies on all‐age cancer patients also show that county‐level measures of unemployment, lower educational attainment, and median household income correspond with greater suicide mortality [11, 12, 13]. We report that this relation also exists in more proximate areas, such as neighborhoods for AYA cancer patients. Inequities in resource distribution within neighborhoods, such as access to social services, mental and physical health care, as well as employment opportunities, may serve as mechanisms by which neighborhood deprivation contributes to suicide among AYA cancer patients. Although we could not directly test these mechanisms, we encourage future work to do so. We also find that non‐Hispanic Black, Hispanic, and Other race/ethnicities have a lower risk of dying by suicide when compared to non‐Hispanic Whites, which coheres with trends seen in the broader US population [45];

On average, AYA cancer patients die by suicide approximately 4.5 years after diagnosis. Comprehensive care that includes mental health services from cancer treatment through survivorship may prove beneficial for this subgroup. Additionally, AYA cancer patients living in Q2 neighborhoods of deprivation show the greatest risk of suicide. We speculate that cancer patients living in Q1 (most deprived neighborhoods) may fall within the social safety net of low‐income governmental programs, whereas those in Q2 may not meet the eligibility requirements for either low‐income programs or private insurance opportunities, contributing to their highest risk of suicide mortality.

Due to the unique medical and psychosocial concerns of AYAs, the National Comprehensive Cancer Network (NCCN) Guidelines for AYA Oncology recommends that all AYAs have access to mental health services for psychiatric symptoms, including anxiety, depression, suicidal thoughts, and self‐injury [46]. This requires referrals to social workers, mental health providers, and community‐based resources, which may be more limited in lower socioeconomic neighborhoods [46]. However, not all AYAs with cancer have access to comprehensive cancer care, particularly those who only receive care from oncology units. In a study that investigated breast cancer mortality based on neighborhood socioeconomic status, women from the most deprived neighborhoods received significantly less NCCN‐concordant guideline treatment compared to women from the most advantaged neighborhoods (78.4% vs. 82.1%) [47]. The shortage of mental health providers in the US, especially those specializing in cancer patient populations, may exacerbate the lack of comprehensive care, with 60% of psychologists reporting having no openings for new patients and nearly 40% reporting that they maintained a waitlist for patients [48]. These obstacles to receiving mental health support may contribute to increased suicide in AYA cancer patients from the most deprived neighborhoods.

In recent years, various approaches have been developed to identify and target patients at a higher risk for suicide. Artificial Intelligence (AI) models have been developed to predict suicidality in clinical populations. AI and other technology‐based interventions may assist with the timely screening and diagnosis of mental health conditions, as the backlog of patients may impede clinical intervention. This may comprise identifying patients at higher risk of suicide using patient demographic and clinical record information [49]. One systematic review of 296 studies showed that AI models successfully identified patients in clinical populations at risk of suicide despite differences in algorithms and implementation [49]. AI models may utilize a patient's address of residence in electronic health records (EHR) to obtain indicators for neighborhood deprivation, therefore identifying those from neighborhoods at a higher risk. However, literature in this area reports that integrating neighborhood socioeconomic status does not predict adverse health (e.g., health service utilization and hospitalizations for accidents, asthma, influenza, myocardial infarction, and stroke) beyond what is already achieved with electronic health records [50]. Further work would benefit from focusing on how integrating neighborhood socioeconomic status into AI models may help identify suicidal behavior in EHR. Given the shortage of mental health professionals and the lack of access to comprehensive mental health care among AYA cancer patients [51], healthcare systems may consider incorporating neighborhood conditions and implementing targeted prevention efforts for high‐risk AYAs living in high‐risk areas.

One cross‐sectional study finds that lower‐income communities have a greater likelihood of having mental health treatment facilities, but have fewer office‐based mental health practices [52]. Although geographic proximity plays a role, other dimensions of accessibility, such as affordability, accommodation, and acceptability, may require attention to sufficiently address mental health needs [52]. More rigorous study designs with longitudinal data may provide information on how changes in the composition of neighborhoods may coincide with changes in mental health services over time. Although telehealth could address geographic gaps in the availability of mental health treatment in low‐income or rural areas, technological challenges and unreliable internet connections may create barriers to care [53, 54].

Our study has limitations. While the neighborhood deprivation index has been widely used in other studies examining changes in cancer‐related outcomes, showing that it captures comprehensive elements of deprivation [55], it may not capture all aspects of the physical and social characteristics of neighborhoods that contribute to adverse mental health or suicidality [17, 18]. We also do not have individual‐level indicators for socioeconomic status, which may attenuate the impact of neighborhood‐level deprivation on suicide mortality [7, 8, 9]. Additionally, our study did not include individual risk factors for suicide, including preexisting mental health conditions, medical financial hardship, or insurance status [28] which may increase the risk of suicide among AYA cancer patients independent of neighborhood deprivation. Future research incorporating such individual‐level factors will provide a comprehensive understanding of risk factors associated with suicide mortality among AYA cancer patients. Lastly, we do not have residential mobility data for AYA cancer patients, as they may have moved to different neighborhoods following diagnosis. Panel data on AYA cancer patients may provide the granularity necessary to assess changes in neighborhood conditions, over time.

Nevertheless, the strengths of our analysis include that our sample comprises 22 cancer registries across the US, excluding Alaska, Illinois, and Massachusetts [37]. This database has the largest geographic coverage available and constitutes more than 40% of the US population over 15 years [37]. Using Cox proportional hazards, our analyses allow us to compare survival among AYAs living in different neighborhood deprivation quintiles. Lastly, the specialized SEER database with neighborhood socioeconomic conditions provides greater granularity to how proximate environments contribute to suicide mortality, as opposed to higher levels of ecology, such as county measures [31]. National mortality files in the US only include county indicators that prevent wide‐scale epidemiologic research on how neighborhood features correspond with suicidality [56].

## 5 Conclusion

Our results demonstrate that neighborhood deprivation corresponds with increased suicide mortality in AYA cancer patients. Due to the unique medical, psychosocial, and financial needs of this population, AYA cancer patients living in deprived neighborhoods should have more consistent screenings to monitor adverse mental health and suicide risk factors from diagnosis throughout the cancer survivorship continuum. The provision of mental health resources, as well as the implementation of EHR‐based suicide risk prediction models in health systems, may prove beneficial to reducing suicide in this high‐risk population.

## Author Contributions

**Abhery Das:** conceptualization, methodology, investigation, writing – original draft, writing – review and editing, visualization, formal analysis, software, data curation, resources, project administration. **Cecily A. Byrne:** conceptualization, investigation, writing – review and editing, validation, methodology, resources. **Vanessa M. Oddo:** investigation, writing – review and editing, visualization, validation, methodology, resources. **Sidra Goldman‐Mellor:** supervision, writing – review and editing, visualization, methodology, validation. **Sage J. Kim:** resources, supervision, writing – review and editing, visualization, methodology, project administration, validation, funding acquisition.

## Disclosure

The authors have nothing to report.

## Ethics Statement

The institution deemed this study exempt from review due to publicly available and de‐identified data.

## Conflicts of Interest

The authors declare no conflicts of interest.

## Supporting information


**Data S1:** cam471247‐sup‐0001‐Supinfo.docx.

## Data Availability

The data that support the findings of this study are openly available in SEER at https://seer.cancer.gov/data/specialized/available‐databases/census‐tract‐request/.
