# Supplementary material for: Neighborhood Deprivation and Suicide Among Adolescent and Young Adult Cancer Patients
Source: Cancer Med. 2025 Sep 26;14(19):e71247. doi: 10.1002/cam4.71247 (PMC12464726; doi:10.1002/cam4.71247)
Supplement: Supplementary file 1 — Data S1: cam471247‐sup‐0001‐Supinfo.docx. [file CAM4-14-e71247-s001.docx]

**Table S1.** Cox proportional hazards model predicting risk of death by suicide or self-inflicted injury as a function of quintiles of neighborhood deprivation with covariates 265,767 among adolescent and young adult (AYA) cancer patients from 22 registries in the United States, 2006-2020.

| **Characteristic** | **Hazard Ratio (95% CI)** |
| --- | --- |
| Neighborhood deprivation |  |
| Quintile 1 (most deprived) | 1.82 (1.14 – 2.90)** |
| Quintile 2 | 1.95 (1.35 – 2.82)*** |
| Quintile 3 | 1.37 (0.94 – 1.98) |
| Quintile 4 | 1.48 (1.05-2.07)** |
| Quintile 5 least deprived) (ref) | -- |
| Race/ethnicity |  |
| NH white (ref) | -- |
| NH Black | 0.48 (0.30 – 0.79)** |
| Hispanic | 0.39 (0.27 0 0.58)*** |
| Other | 0.77 (0.51 – 1.17) |
| Sex |  |
| Female (ref) | -- |
| Male | 2.83 (2.18 – 3.67)*** |
| Age group |  |
| 15-19 (ref) | -- |
| 20-24 | 1.10 (0.63 – 1.90) |
| 25-29 | 1.01 (0.60 – 1.73) |
| 30-34 | 1.08 (0.64 – 1.82) |
| 35-39 | 1.84 (1.12 – 3.04)** |
| Relationship status |  |
| Single (never married) (ref) | -- |
| Married (including common law) | 0.62 (0.47 – 0.82)** |
| Other | 1.50 (0.99 – 2.25) |
| Stage |  |
| In situ/Localized (ref) | -- |
| Regional | 1.08 (0.82 – 1.43) |
| Distant | 1.46 (0.99 – 2.16) |
| AYA site |  |
| Carcinomas (ref) | -- |
| Leukemias and related disorders | 0.79 (0.44 – 1.41) |
| Lymphomas | 0.81 (0.54 – 1.22) |
| CNS^1^ and intracranial and intraspinal neoplasms | 1.12 (0.63 – 1.99) |
| Sarcomas | 0.68 (0.34 – 1.36) |
| Blood and lymphatic vessel tumors | 0.77 (0.19 – 3.15) |
| Nerve sheath tumors | 1.78 E-19 |
| Gonadal and related tumors | 0.97 (0.67 – 1.39) |
| Melanomas | 0.88 (0.56 – 1.38) |
| Miscellaneous specified neoplasms | 1.10 (0.15 – 7.92) |
| Unspecified malignant neoplasms | 2.02E-19 |
| Persistent poverty |  |
| No (ref) | -- |
| Yes | 0.98 (0.61 – 1.59) |
| Urbanicity |  |
| Urban (ref) | -- |
| Rural | 1.04 (0.73 - 1.50) |

*p<0.01, **p<0.05, ***p<0.001

^1^Central nervous system

**Table S2.** Proportionality assumption tests for each variable and for the overall model in the survival analysis of suicide or self-inflicted injury mortality among adolescent and young adult (AYA) cancer patients from 22 registries in the US, 2006-2020.

| Variable | p-value* |
| --- | --- |
| Neighborhood deprivation | 0.62 |
| Urbanicity | 0.76 |
| Sex | 0.07 |
| Race/ethnicity | 0.41 |
| Stage | 0.25 |
| Relationship status | 0.67 |
| Age group | 0.14 |
| AYA site | 0.22 |
| Persistent poverty | 0.40 |
| Overall model | 0.16 |

*p>0.05 indicates that it does not violate the proportionality assumption

**Figure S1.** Proportional hazards assumption plot (Schoenfeld method*) of neighborhood deprivation in the survival analysis of suicide or self-inflicted injury mortality among adolescent and young adult (AYA) cancer patients from 22 registries in the US, 2006-2020.


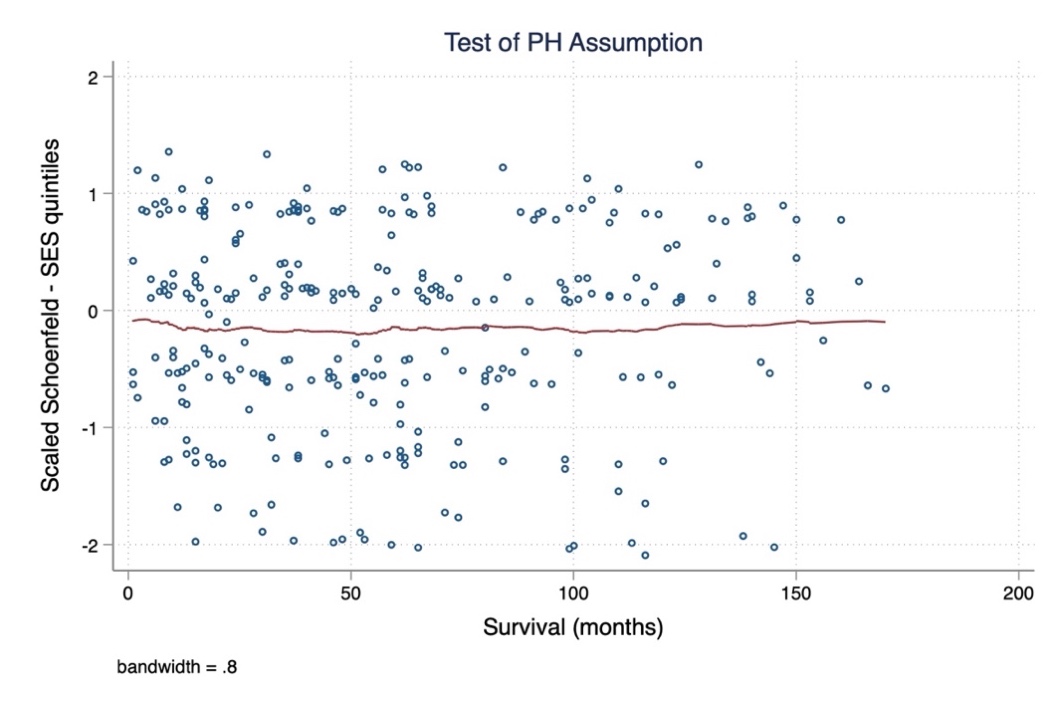


*Parallel line indicates that the proportionality assumption is not violated

**Table S3.** Cox proportional hazards model predicting risk of death by suicide or self-inflicted injury (compared to those who are still alive) as a function of quintiles of neighborhood deprivation among 265,767^1^ adolescent and young adult (AYA) cancer patients from 22 registries in the United States, 2006-2020.

| **Characteristic** | **Hazard Ratio (95% CI)** |
| --- | --- |
| **Neighborhood deprivation** |  |
| Quintile 1 (most deprived) | 1.82 (1.14 – 2.90)** |
| Quintile 2 | 1.95 (1.35 – 2.82)*** |
| Quintile 3 | 1.37 (0.94 – 1.98) |
| Quintile 4 | 1.48 (1.05 – 2.07)** |
| Ref: Quintile 5 (least deprived) | -- |

**p<0.05, ***p<0.001

^1^ Adjusted for individual and neighborhood-level covariates

**Figure S2.** Survival probability of death by suicide or self-inflicted injury among adolescent and young adult (AYA) cancer patients by neighborhood urbanicity from 22 registries in the United States, 2006-2020.


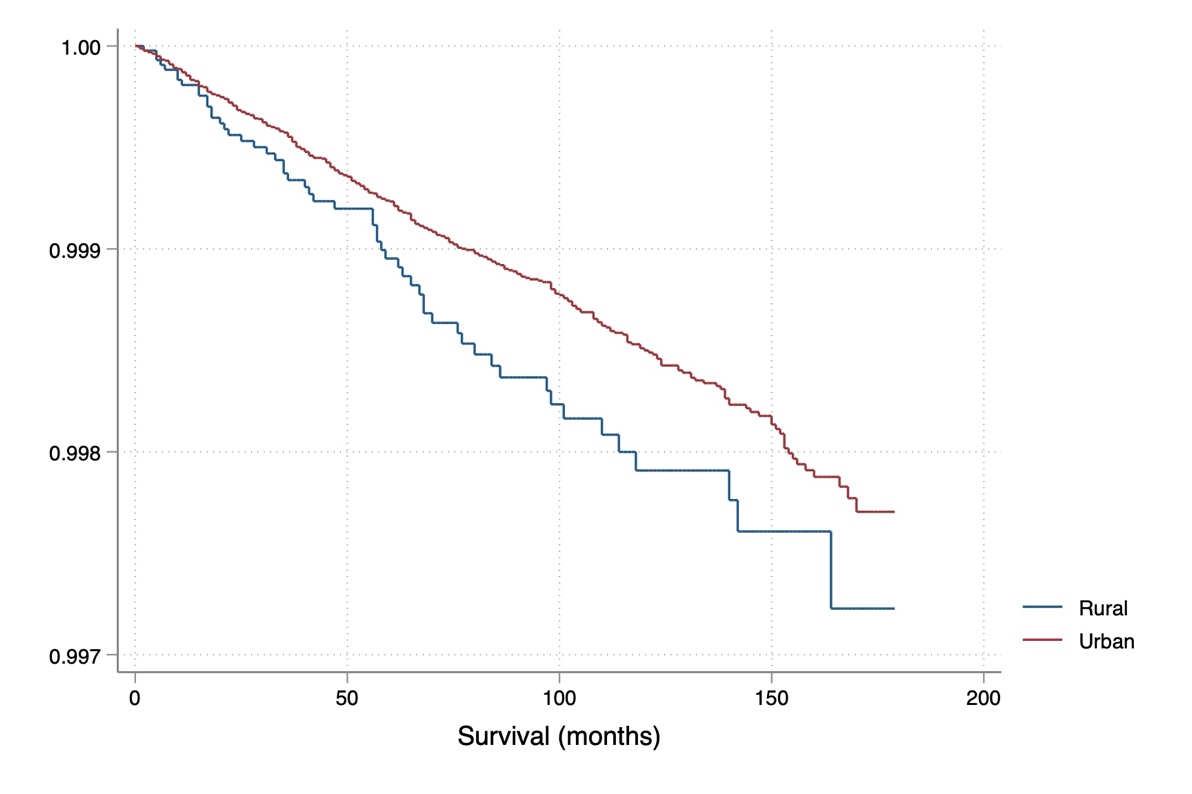


*Log-rank: p<0.25

**Figure S3.** Survival probability of death by suicide or self-inflicted injury among adolescent and young adult (AYA) cancer patients by sex from 22 registries in the United States, 2006-2020.


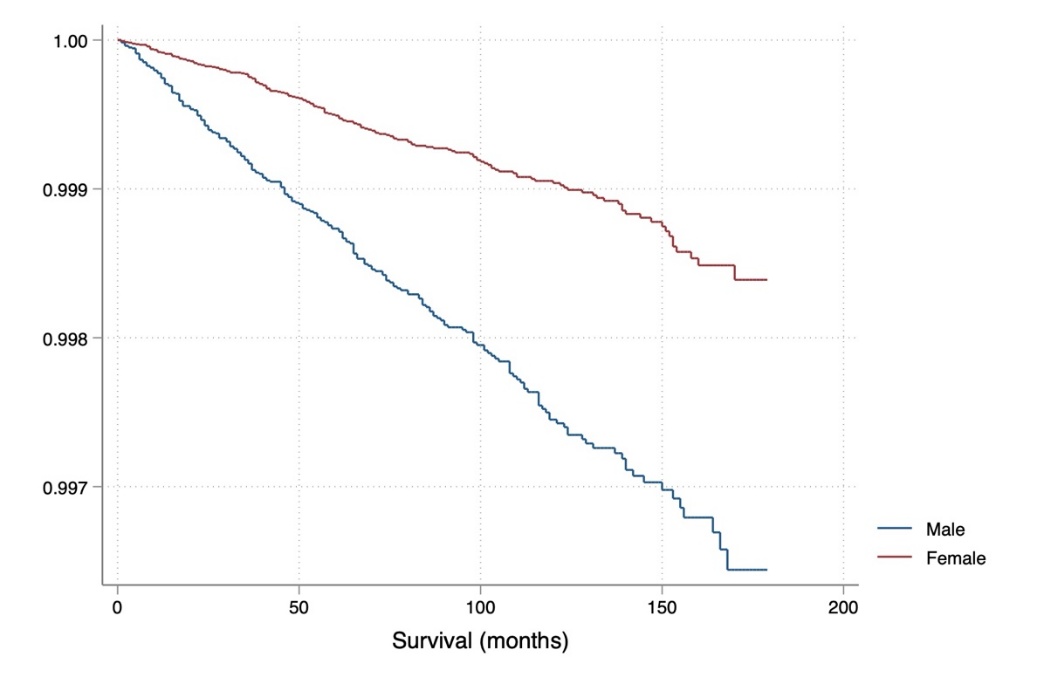


*Log-rank: p<0.25

**Figure S4.** Survival probability of death by suicide or self-inflicted injury among adolescent and young adult (AYA) cancer patients by race/ethnicity from 22 registries in the United States, 2006-2020.


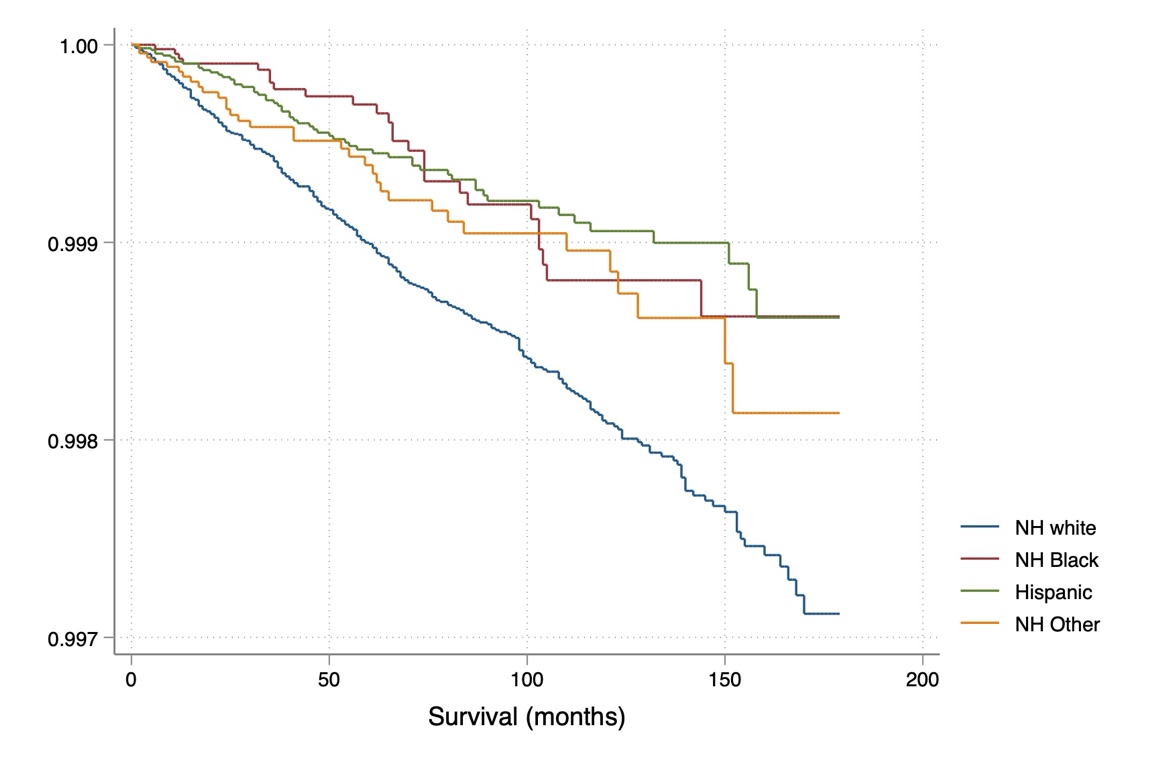


*Log-rank: p<0.25

**Figure S5.** Survival probability of death by suicide or self-inflicted injury among adolescent and young adult (AYA) cancer patients by stage from 22 registries in the United States, 2006-2020.


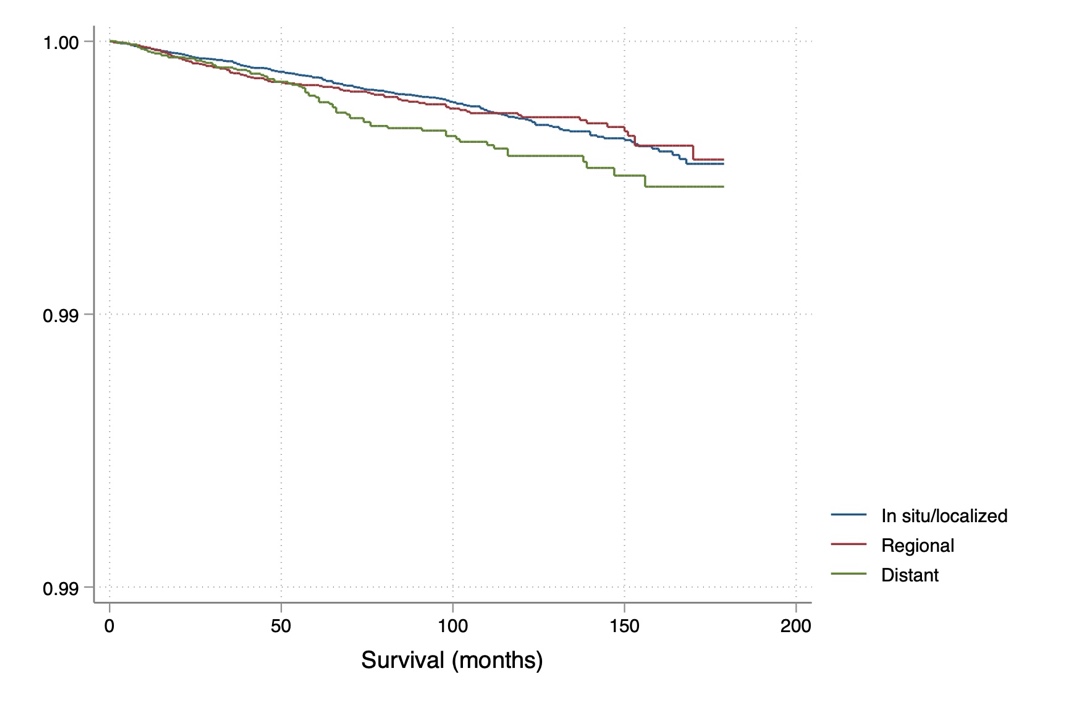


*Log-rank: p<0.25

**Figure S6.** Survival probability of death by suicide or self-inflicted injury among adolescent and young adult (AYA) cancer patients by relationship status from 22 registries in the United States, 2006-2020.


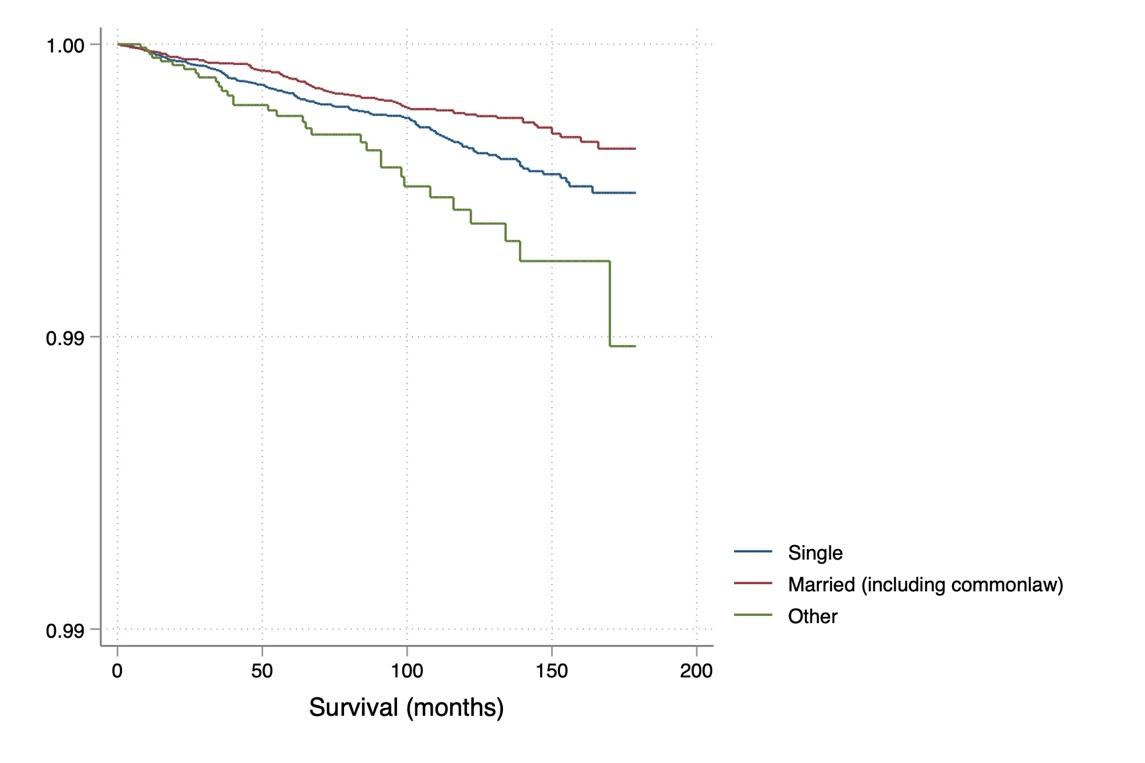


*Log-rank: p<0.25

**Figure S7.** Survival probability of death by suicide or self-inflicted injury among adolescent and young adult (AYA) cancer patients by AYA cancer site from 22 registries in the United States, 2006-2020.


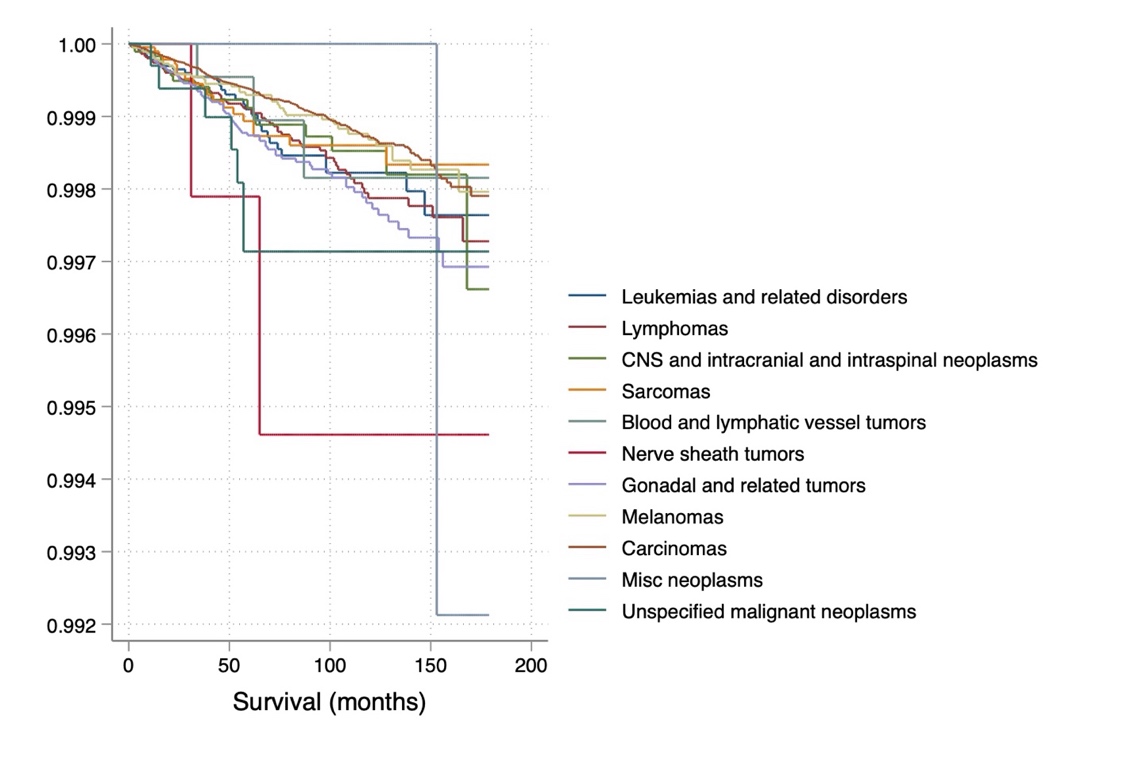


*Log-rank: p<0.25

**Figure S8.** Survival probability of death by suicide or self-inflicted injury among adolescent and young adult (AYA) cancer patients by persistent poverty from 22 registries in the United States, 2006-2020.


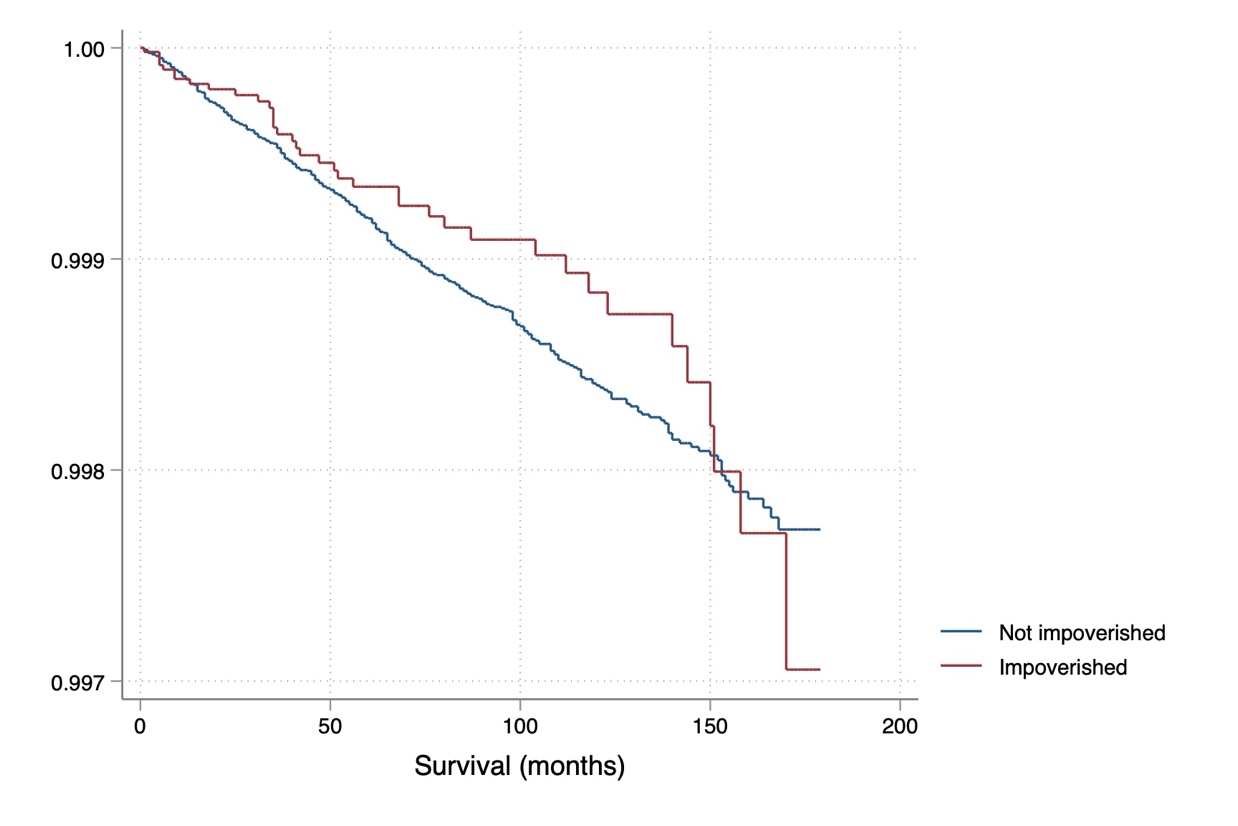


*Log-rank: p>0.25
